# Recent Progress in Lyme Disease and Remaining Challenges

**DOI:** 10.3389/fmed.2021.666554

**Published:** 2021-08-18

**Authors:** Jason R. Bobe, Brandon L. Jutras, Elizabeth J. Horn, Monica E. Embers, Allison Bailey, Robert L. Moritz, Ying Zhang, Mark J. Soloski, Richard S. Ostfeld, Richard T. Marconi, John Aucott, Avi Ma'ayan, Felicia Keesing, Kim Lewis, Choukri Ben Mamoun, Alison W. Rebman, Mecaila E. McClune, Edward B. Breitschwerdt, Panga Jaipal Reddy, Ricardo Maggi, Frank Yang, Bennett Nemser, Aydogan Ozcan, Omai Garner, Dino Di Carlo, Zachary Ballard, Hyou-Arm Joung, Albert Garcia-Romeu, Roland R. Griffiths, Nicole Baumgarth, Brian A. Fallon

**Affiliations:** ^1^Icahn School of Medicine at Mount Sinai, New York, NY, United States; ^2^Department of Biochemistry, Fralin Life Sciences Institute, Virginia Tech, Blacksburg, VA, United States; ^3^Lyme Disease Biobank, Portland, OR, United States; ^4^Tulane University Health Sciences, New Orleans, LA, United States; ^5^Institute for Systems Biology, Seattle, WA, United States; ^6^State Key Laboratory for the Diagnosis and Treatment of Infectious Diseases, The First Affiliated Hospital, Zhejiang University School of Medicine, Hangzhou, China; ^7^Division of Rheumatology, Department of Medicine, Lyme Disease Research Center, Johns Hopkins University School of Medicine, Baltimore, MD, United States; ^8^Cary Institute of Ecosystem Studies, Millbrook, NY, United States; ^9^Department of Microbiology and Immunology, Virginia Commonwealth University Medical Center, Richmond, VA, United States; ^10^Bard College, Annandale, NY, United States; ^11^Department of Biology, Northeastern University, Boston, MA, United States; ^12^Yale University School of Medicine, New Haven, CT, United States; ^13^Department of Clinical Sciences, Comparative Medicine Institute, College of Veterinary Medicine, North Carolina State University, Raleigh, NC, United States; ^14^Department of Microbiology and Immunology, Indiana University School of Medicine, Indianapolis, IN, United States; ^15^Steven & Alexandra Cohen Foundation, Stamford, CT, United States; ^16^University of California, Los Angeles, Los Angeles, CA, United States; ^17^Department of Psychiatry and Behavioral Sciences, Johns Hopkins University School of Medicine, Baltimore, MD, United States; ^18^Center for Immunology and Infectious Diseases and the Department of Pathology, Microbiology & Immunology, School of Veterinary Medicine, University of California, Davis, Davis, CA, United States; ^19^Columbia University Irving Medical Center, New York, NY, United States

**Keywords:** Lyme disease, pathogenesis, diagnosis, treatment, prevention, field building, PTLD, vaccine

## Abstract

Lyme disease (also known as Lyme borreliosis) is the most common vector-borne disease in the United States with an estimated 476,000 cases per year. While historically, the long-term impact of Lyme disease on patients has been controversial, mounting evidence supports the idea that a substantial number of patients experience persistent symptoms following treatment. The research community has largely lacked the necessary funding to properly advance the scientific and clinical understanding of the disease, or to develop and evaluate innovative approaches for prevention, diagnosis, and treatment. Given the many outstanding questions raised into the diagnosis, clinical presentation and treatment of Lyme disease, and the underlying molecular mechanisms that trigger persistent disease, there is an urgent need for more support. This review article summarizes progress over the past 5 years in our understanding of Lyme and tick-borne diseases in the United States and highlights remaining challenges.

## Introduction

According to the Center for Disease Control and Prevention (CDC), the number of vector-borne diseases reported to the National Notifiable Diseases Surveillance System (NNDSS) between 2004 and 2016 reached a total of 642,602 cases. Of these, tick-borne diseases (TBDs) accounted for 77% (491,671 cases) of reported cases with the total number of cases doubling in 13 years. The pace of emergence of new tick-borne disease cases increased not only for Lyme disease (LD), the most predominant TBD with 82% of cases, but also for spotted fever rickettsiosis, babesiosis, anaplasmosis, and Powassan disease (1). In this review, we highlight the major scientific advances made primarily in the field of LD research in the United States (USA).

LD, also known as Lyme borreliosis, is a growing health problem in the USA. LD is caused by pathogenic species in the *Borreliella* genus (for the relationship with the *Borrelia* genus, see section Genomic Insights From Borreliaceae Lineages). These spirochetal bacteria are transmitted from vertebrate reservoirs to human hosts through bites from infected *Ixodes spp*. ticks. *Borreliella burgdorferi (B. burgdorferi*, hereafter *Bb)* is the most common agent of LD in the USA (1). The CDC recently estimated ~476,000 clinician diagnosed cases of LD every year in the USA based on insurance claims data from 2010 to 2018 (2), a significant increase from their previous estimate of ~329,000 annual cases using similar methods to generate data from 2005 to 2010 (3). If untreated, infection with *Bb* can lead to health problems affecting the skin, joints, nervous system, or less commonly, the heart (4). While most individuals return to health following antibiotic treatment for LD, others go on to experience chronic health problems that can last months to years. One well-defined clinical subgroup of LD patients who experience ongoing symptoms following treatment is Post-treatment Lyme disease (PTLD) (see section 2.3.2 PTLD). Medical costs related to LD and PTLD are estimated to be between $712M−1.3B each year in the USA (5). The causes of PTLD are not yet well-understood but are an active area of research due to their critical importance to advancing therapy development and effective treatment for this patient population. The two most salient hypotheses for etiology of PTLD include persistence of infection or antigenic debris, persistence of inappropriate immune activation and inflammation, or some combination of these (see section Pathogenesis below). The research community has largely lacked the necessary funding to properly advance scientific and clinical understanding of LD and its sequelae, and to develop and evaluate new approaches for prevention, diagnosis, and treatment. The annual NIH investment in LD research so far has been small compared to many other infectious diseases (see Table 1) (6).

**Table 1:** NIH support for LD research is currently low compared to other infectious diseases.

**Disease**	**NIH Funding FY 2018 (in millions)^a^**	**USA reported cases in 2018**	**Funding per reported case in 2018**
HIV/AIDS	$2,995	36,400^b^	$82,280
Malaria	$202	~2,000^c^	~$101,000
West Nile Virus	$36	2,647^d^	$13,600
Lyme Disease	$30	~33,666^e^ (~476,000 estimated cases)^g^	~$891 ($63 per estimated case)
Tuberculosis	$403	9,029^f^	$44,634

*For purposes of consistency and comparison across diseases, the table uses funding per case based on the number of reported cases. A difference sometimes exists between the reported number of cases per year and estimates of the actual incidence in some infectious diseases. For LD, the difference is more than 10-fold. For example, the number of reported cases in the USA in 2018 is ~34 k while the estimated number of annual cases is ~476 k (g). Therefore, the research investment by the NIH for LD is around $63 per new estimated case in 2018. This table is adapted and updated from a version in the Tick-borne Diseases Working Group (TBDWG) report to Congress (6)*.

a *https://report.nih.gov/categorical_spending.aspx*

b *https://www.hiv.gov/hiv-basics/overview/data-and-trends/statistics*

c *https://www.cdc.gov/parasites/malaria/index.html*

d *https://www.cdc.gov/westnile/statsmaps/cumMapsData.html*

e *https://wonder.cdc.gov/nndss/static/2018/annual/2018-table2i.html*

f *https://www.cdc.gov/mmwr/volumes/68/wr/mm6811a2.htm*

g *https://www.cdc.gov/lyme/stats/humancases.html*

Considering the rapid growth in prevalence of LD and the risk for significant long-term health consequences of those infected (7), a multifaceted effort is needed to create better prevention practices, diagnostics, and treatments, along with advancing basic science about ticks, tick-borne pathogens, and the pathophysiology of LD. In this review, we summarize key advances in each of these areas over the past 5 years and identify challenges and opportunities for the field. We aim to highlight many important studies, but due to space constraints, we are not able to discuss all relevant publications. Where available, we also reference more in-depth review articles on specific topics. While LD is a global public health concern across the Northern Hemisphere, this review article is largely USA-centric in terms of manuscripts discussed since aspects of LD vary geographically, including the primary causative agents and their vectors; pathogenicity and common disease manifestations; and the prevalence and incidence of disease.

## Clinical and Translational Medicine

Next, we review progress in the diagnosis and treatment of LD, including emerging diagnostic assays and novel therapies. We also describe 2-well-defined subgroups of patients with post-treatment sequelae, including those with antibiotic-refractory Lyme arthritis (LA) and PTLD.

### Diagnosis

The diagnosis of LD can be a complex task for the provider because, outside of the erythema migrans (EM) lesion of early LD, diagnosis relies on non-specific clinical signs and symptoms that may or may not be supported by laboratory evidence. A prospective study evaluated the ability of emergency room (ER) physicians across 5 hospitals in endemic areas in the Northeast of the USA to accurately discriminate between LD (early disseminated or late) and non-LD using clinical judgment alone, prior to the receipt of laboratory evidence. Among 1,021 children being evaluated for LD (based on presence of one or more EM lesions or Lyme serology tests ordered and compatible symptoms) and enrolled in the study between 2015 and 2017, clinician suspicion of LD in the ER setting was found to be minimally accurate compared to diagnoses supported by laboratory evidence. Twelve percent of patients whom the treating clinician deemed to be unlikely to have LD, actually had LD. Thirty-one percent of patients whom the clinician deemed very likely to have LD, actually did not have LD (8). A true case of early disseminated or late LD was defined in this study as those with compatible symptoms and positive two-tier serology per guidelines, the limitations of which are reviewed below. The accuracy of clinician assessment of patients presenting with a single EM lesion (*n* = 42) was not assessed. The challenges of discriminating between an EM lesion of LD and a non-EM lesion are included below, along with a description of a novel imaging tool that may aid clinician assessment of this sign of LD (9).

Many patients struggle with getting a timely diagnosis and treatment for LD. Around 40% of patients diagnosed with LD have signs and symptoms associated with disseminated or late LD, indicating that delayed diagnosis and treatment are a common occurrence (10). In a recent population-based study of 778 patients surveyed in Pennsylvania who were treated for LD in the past 5 years, 31% had a time to treatment >30 days and 10% had time to treatment >6 months, where time to treatment is defined as the sum of time to first medical contact and time under care until receiving treatment (11). A qualitative study of 26 patients treated for LD in Pennsylvania suggests that patient appraisal of their own signs and symptoms plays a role in delayed treatment, specifically the misattribution of non-specific symptoms, the intermittent nature of symptoms and the lack of a “bull's-eye rash,” which is commonly misunderstood to be the only representative skin lesion of LD (12). High rates of initial delayed or misdiagnosis is also commonly reported by LD patients that meet the PTLDS case definition (13) or those with chronic symptoms more broadly (14).

The consequences of the diagnostic challenges in LD are potentially significant for patients and may lead to missed or delayed diagnosis and exposure to inappropriate or inadequate treatment. Next, we review some recent findings and the current challenges related to the diagnosis of LD.

#### Exposure to Ticks

The collection of tick exposure history from patients suspected of having LD lacks sensitivity because ticks are stealth biters. They are able to avoid detection by human hosts during feeding. Many people diagnosed with LD have no recollection of being bitten by a tick (15). While the major endemic regions in the USA are the Northeast (16), mid-Atlantic and upper Midwest states, *Ixodes* spp. ticks capable of carrying LD pathogens are found in many states. A recent citizen-science based effort to collect ticks submitted by volunteers from across the USA identified ticks capable of carrying *Borreliella* species in 35 states (17). In California, where LD is not considered endemic, infected *Ixodes spp*. ticks have been found in 42 counties (72%) according to surveillance data (18).

#### Serological Testing

Two-tiered serological testing is widely used to support the diagnosis of LD. The two-tiered testing algorithm consists of a first-tier enzyme immunoassay (EIA) or ELISA, and for samples that are positive or equivocal (borderline) on the first tier, a second tier immunoblot is performed. Using the CDC's algorithm, the immunoblot is positive if at least 2 of 3 bands are present on the IgM immunoblot within 30 days of symptom onset or 5–10 bands are present on the IgG immunoblot at any time (19). A modified two-tiered testing algorithm was approved by the CDC in 2019, which uses a first-tier ELISA, and instead of the confirmatory immunoblot, uses 1 or 2 additional ELISAs that target different antigens than the first-tier ELISA (20).

Serology presents several challenges for accurate diagnosis. The human body takes time to generate anti-*Bb* antibodies, so serological testing is not sensitive during early infection (21–24)—the period when treatment is most likely to succeed. For patients with disseminated LD or later manifestations such as late Lyme arthritis, serological testing has improved performance compared to early disease (22, 25, 26). However, current tests also lack sensitivity following antibiotic treatment of acute LD, as seroconversion occurs less frequently (21, 23, 27–30). Rebman et al. ran two-tier serology on acute and convalescent sera samples collected from 104 patients with clinician-diagnosed EM rash and 21 days of antibiotic treatment. They observed 41 (39.4%) of these patients were seronegative at both the acute and convalescent time points; only 7 (6.7%) patients were observed to have IgG seroconversion at either time point (29). Seroconversion was also rare in samples from the Lyme Disease Biobank, where only 3 of 83 samples (3.6%) from patients with EM > 5 cm seroconverted at the convalescent draw (2–3 months after the acute draw) (23). Other widely known limitations of serological tests are that they are unable to distinguish between a prior exposure to *Bb* and an active infection, may cross-react with non-*Bb* antibodies, are subject to variable results depending the selection of antigens used in the first-tier test (31) and some assays, especially the Western immunoblot, require interpretation that may introduce bias (32, 33).

For patients with an EM > 5 cm in an endemic area with a history of tick exposure, a clinical diagnosis is sufficient (34). Testing is not indicated in these patients, and the serologic tests would likely be negative due to lack of antibody development in early disease. For patients with early LD presenting without EM, diagnosis is incredibly challenging.

#### Signs and Symptoms

If untreated, a patient with a *Bb* infection may go through several stages of LD, with different signs and symptoms at each stage [reviewed in (35)]. In most people, the first stage of LD begins with “flu-like” symptoms and an EM lesion. LD is known as the “great imitator” because symptoms are varied and often overlap with common health complaints, sometimes making early diagnosis more difficult (36, 37). The most common symptoms of early LD are fever, chills, headache, fatigue, neck stiffness, myalgia, joint pain and swollen lymph nodes. There are likely hundreds of health conditions with significant overlap with these non-specific signs and symptoms. As spirochetes disseminate from the site of the tick bite, additional EMs and manifestations can occur including 7th cranial nerve palsy, meningitis, or Lyme carditis [reviewed in (38)]. In the third stage, without proper treatment, patients may also experience neuroborreliosis or Lyme arthritis (LA).

The type and severity of LD manifestations are known to vary across infected individuals for reasons that are unclear but are likely attributable to both, differences in the infecting pathogen and the characteristics of the infected individual. They range from asymptomatic or subclinical infection (39–41) all the way to severe complications from LD that, in rare cases, result in death from Lyme carditis (42, 43).

#### EM Lesion

The characteristic EM lesion develops inconsistently across humans 3–30 days following a bite from an infected tick (44). The EM is often an annular, erythematous, expanding cutaneous lesion that may or may not have a central clearing. While it is sometimes referred to as a “bulls-eye rash,” presentation is known to vary considerably (15, 45–47). Variation in skin pigmentation, as well as coloring and shape of the rash may also lead to missed or delayed clinical diagnoses (48). The central clearing in the rash is reported to be less common in endemic areas compared to non-endemic areas (15). While reports vary across studies, up to 30% of individuals diagnosed with LD do not develop an EM lesion (47, 49–52) or its presence is missed. If an EM lesion is absent, there is no clinically recommended laboratory test available to aid in the diagnosis of early LD because the currently recommended serologic tests are highly insensitive in the first few weeks of infection (51).

#### Direct Detection of Bb

Many bacterial infections are diagnosed using a variety of culture methods and the confirmation of pathogen identity through molecular techniques or differential biochemical assays (53, 54). This is not currently practical or feasible for LD. The direct detection of the pathogen in blood can be a challenge because of the narrow window of spirochetemia that is more likely during early infection and the low numbers of circulating *Bb* (55, 56). While the pathogen may disseminate from the site of the tick bite through the blood, it also disseminates through the lymphatics and is known to invade other more privileged tissues, such as the heart, nervous system, and connective tissue. *Bb* is a fastidious, slow-growing bacteria that requires up to 12 weeks of incubation in culture before a negative result is determined, which is too long to be useful in clinical diagnosis (32). In one study on the ability to detect spirochetemia in patients with EM through culture methods, they estimated 1 cultivable spirochete per 10 mL of whole blood (55). *Bb* culture also requires specialized skills and tools that most laboratories are not equipped with outside of the research setting, where these techniques remain valuable for basic science research (21). Finally, antibiotic treatment decreases culture positivity rates, making it useful only in untreated patients (21). Blood, serum or plasma is not a reliable tissue to detect *Bb* by PCR because the spirochetes are transient and in low copy number. Skin biopsy from the EM lesion is a more useful tissue diagnostically, but this step is invasive and patients that present with an EM lesion do not require laboratory confirmation for diagnosis of LD (21).

#### Emerging Diagnostics

For the reasons outlined above, there is an urgent need for pathogen-detection methods that are highly sensitive and specific and capable of reliably detecting infection by multiple pathogenic species of *Borreliella* and strains of *Bb* (see section Genomic Insights From Borreliaceae Lineages) at all stages of infection and disease (57). Of special concern are individuals with acute infection that do not present with an EM rash and have yet to generate a humoral response to *Bb*. The factors that control the development of EM rash also need to be delineated, along with a surrogate set of biomarkers to aid diagnosis of more complex cases of suspected LD. *Ixodes* ticks can carry multiple human pathogens (see section Transmission of *Bb* via Ixodes spp. Vectors), and diagnostic methods capable of detecting the most prevalent tick-borne pathogens and clinically relevant strains of *Borreliella* are needed. Promising new diagnostic methods are being developed using serology, direct detection assays, and other tests that measure host response to the pathogen. Select assays are outlined in Table 2. There is also a significant unmet need for diagnosing PTLD. Currently, this diagnosis is performed, in part, based on self-reporting of symptoms. Several groups are investigating correlation between blood transcriptome; blood metabolome; and gut microbiome of PTLD patients in search of potential diagnostic and causal markers. Of note are recent studies describing a distinct microbiome signature (58) and a blood metabolome signature (59).

**Table 2:** Emerging diagnostic assays for LD.

**Test**	**Description**	**References**
**Serological assays**		
TBD-Serochip	Array assay that discriminates antibody response to 8 tick-borne pathogens	(60)
mChip-Ld	Multiplex microfluidics assay targeting 3 Bb antigens for POC use	(61)
xVFA	Multiplex paper based lateral flow assay targeting 7 Bb antigens for POC use	(62)
**Direct detection assays**		
Karius test	Microbial cell-free DNA unbiased metagenomic sequencing	(63, 64)
PCR-ESI/MS	Direct detection PCR with electrospray ionization mass spectrometry	(24, 65)
Nanotrap urine	Nanotrap assay to measure *Bb* OspA c-terminus peptide	(66)
**Host-focused assays**		
Transcriptome	Transcriptome profiling by RNA-Seq to identify gene expression signature	(67, 68)
Proteome	Targeted mass spectrometry-based approach to identify protein biomarkers	(69)
SERA	Antibody repertoire analysis to identify Lyme specific motifs	(70)
ImmunoSEQ	T-cell receptor sequencing to identify Lyme specific signature	(71) ^2^
Microbiome	Microbiome sequencing to identify Lyme specific signature	(58)
Metabolomics	LC-MS/LC-MS-SRM to analyze small molecule metabolites to develop biosignature	(59, 72–75)
QuantiFERON-Lyme	Assay that measures IFN-γ release from T-cells in response to *Bb*	(76)
**Imaging**		
Image Analysis	Deep learning algorithms that discriminate between EM and other skin lesions	(9, 77)
HS-198	A small-molecule fluorescent conjugate that targets a conserved protein in Bb for *in vivo* imaging.	(78)

*Bb, B. burgdorferi; POC, Point of care; LC-MS, Liquid chromatography-mass spectrometry*.

### Treatment

#### Recommended Treatment

Antimicrobial therapy for LD is often successful, especially when patients are treated in the early phase following detection of an EM lesion (79). As disease progresses, treatment must be extended and may be less effective (80, 81). Administration of doxycycline or amoxicillin for 14–21 days is the recommended treatment for early or early disseminated phase patients who do not have neurological involvement (44). Lyme arthritis is an indication of disseminated disease and the recommended treatment for this is the aforementioned oral antibiotic for 28 days. For patients with clinically evident neurological involvement, treatment with intravenous ceftriaxone is recommended. These suggested regimens are based on objective measurements. However, in the guidelines from the Infectious Disease Society of America (IDSA), the authors point out that “Response to treatment is usually slow and may be incomplete” (82).

There is widespread agreement in the medical community about the appropriate treatment of acute LD (83), however the appropriate treatment of patients meeting the Post-treatment Lyme disease syndrome (PTLDS) case definition remains a challenge due to incomplete knowledge about the condition and related uncertainties. Moreover, the recently updated IDSA guidelines for the treatment of LD remove mention of PTLDS altogether (83).

More clinical trials are needed to assess the efficacy and safety of drug regimens and complementary therapies for LD and its sequelae. This year, the first clinical trial coordinating center was established at Columbia University to facilitate the conduct of high-quality multi-site clinical trials and pilot studies related to LD and other TBDs.^1^ Furthermore, tools capable of discriminating the etiology of persistent health issues following treatment for LD are needed in order to improve therapy development efforts and target treatments for more precise patient-centered care.

#### Drug Discovery and Preclinical Studies

Although early-stage LD can be successfully treated with doxycycline or amoxicillin, late-stage LD with arthritis and neurological symptoms can be refractory to antibiotic treatment. Wu et al. showed that stationary phase *Bb* are unexpectedly susceptible to cell wall synthesis inhibitors, and vancomycin in particular almost completely eradicates persisters *in vitro* (84) (see section Persistent Infection). Feng et al. recently identified FDA-approved drug candidates that are more effective at killing dormant *Bb* persisters *in vitro* than the current Lyme antibiotics (85).They found the drug combination Daptomycin + Cefoperazone (or Cefuroxime) + Doxycycline was most effective at eradicating *Bb* grown *in vitro* and more recently in the mouse model for *Bb* persistence (86, 87).

#### Complementary Therapies

Zhang et al. recently identified natural products and botanical medicines (*Cryptolepis sanguinolenta* (Yellow dye root)*, Juglans nigra* (Black walnut), *Polygonum cuspidatum* (Japanese knotweed), *Uncaria tomentosa* (Cat's claw), *Artemisia annua* (Sweet wormwood), *Cistus incanus*, and *Scutellaria baicalensis* (Chinese skullcap) with good activity against both stationary phase and growing *Bb* (88). More recently, botanical medicines *Cryptolepis sanguinolenta, Artemisia annua, Scutellaria baicalensis, Polygonum cuspidatum*, and *Alchornea cordifolia* have also been shown to have activity against *Babesia duncani* (89). Since the above herbal medicines that have activity against *Bb* and *Babesia* have been used traditionally in patients with good safety profile, proper clinical trials are needed to evaluate their utility in treating patients with LD and coinfections. In addition, some essential oils such as oregano, cinnamon bark, clove bud, citronella, garlic, allspice, myrrh, hydacheim, and Litsea cubeba were shown to have excellent activity against both stationary phase and growing *Bb* (88, 90). However, further studies are needed to evaluate the active compounds in the essential oils, in order to elucidate the specific activity, to assess their potential toxicity and pharmacokinetic properties for activity against *Bb* infection in animal models before human use.

### Well-Defined LD Patient Subgroups With Posttreatment Sequelae

Antimicrobial therapy effectively treats LD in most people, however some patients experience ongoing health issues following treatment (4, 91). Antibiotic-refractory LA is the most well-characterized subgroup of patients with posttreatment sequelae. In recent years, characterization of patients with PTLD, a well-defined subset of patients with persistent or chronic LD, has also advanced considerably. Next, we review progress in the description of each of these post-treatment sequelae.

#### Antibiotic Refractory LA

The LD patient subgroup with LA has been meticulously studied for decades. In 1975, a cluster of cases was brought to the attention of the Connecticut State Department of Health independently by two mothers concerned about the number of children with swollen knees with similarities to juvenile arthritis, a rare condition. An investigation revealed 51 similar cases (39 of these children) in Old Lyme, Connecticut and adjacent townships (92). By 1983, the causative agent and vector of LD were identified (93). LA—the proliferative and persistent synovitis of one or more large joints—is the most prevalent symptom in late-stage LD patients [reviewed in (94)]. LA may wax and wane months to years following untreated infection (95). Around 10% of LA patients experience persistent LA that does not resolve within a couple months after one or more rounds of antibiotic therapy (95–97). LA patients refractory to oral antibiotics are treated with intravenous ceftriaxone antibiotic therapy, with mixed results in adults (97, 98) and children (99). Persistent LA despite aggressive treatment with antibiotics is referred to as antibiotic refractory LA (100), or if no evidence of ongoing infection, post-infectious LA (101). Among patients with persistent Lyme arthritis, determining whether persistent joint inflammation is due to an ongoing antibiotic-refractory infection or a post-infectious immune response also raises important treatment dilemmas that are not yet resolved, as reviewed elsewhere (95). The true cause of antibiotic-refractory LA has remained enigmatic. Recent developments, however, suggest a complex interplay between both bacterial and host factors (102).

What bacterial components may contribute to inflammation after therapy? While the debate surrounding persistent infection continues (see section Persistence), undetectable levels of bacteria in the SF of some patients with antibiotic refractory LA has led to a search for alternative explanations for persistent inflammation. Intravital microscopy studies of antibiotic treated mice observed the persistence of *Bb*-derived material (103), which precipitated the possibility that bacterial debris may be responsible for immune activation. Later, we highlight a recent finding that a component of the bacterial cell envelope may exacerbate and prolong the initial response to the LD agent (see section Persistent Antigenic Debris).

#### PTLD

PTLD is a narrow but defined subset of patients with persistent symptoms following treatment for LD [reviewed in (30, 44)]. Patients with PTLD suffer persistent or relapsing symptoms, such as severe fatigue, cognitive issues, sleep disturbance, and musculoskeletal pain that negatively impact their functional abilities at least 6 months following treatment for LD. The reductions in quality of life for patients with PTLD are comparable to patients suffering from congestive heart failure (104, 105). These health problems can last months or years following the initial treatment for LD. The incidence of PTLD is as high as 20% in some patient populations (13, 44, 91, 106–108). Prevalence of PTLD is currently difficult to ascertain, due in part to the variability in the case definition applied in the literature and variability in reported rates of treatment failures among patients with LD, so estimates range from 69,000 to more than 1 million cases in the USA (107, 109).

Descriptions of patients with persistent symptoms following antibiotic treatment for LD date back to studies in the 1980's (110, 111) and 1990's (79, 80, 112, 113). The term “post-treatment [chronic] Lyme disease syndrome” and a case definition PTLDS were first described in 2003 (114) and 2006 (44), respectively. Today this case definition, along with standardized measurement of subjective symptoms (115), informs the eligibility and exclusion criteria for many studies and trials related to the broader and more heterogeneous population with chronic or persistent Lyme (91, 116, 117), so that outcomes from research studies may be compared and potential mechanisms of pathogenesis defined with less ambiguity (44). Recent studies have deployed deep phenotyping methods and identified multiple biomarkers that distinguish these patients from LD patients that return to health and/or controls, as described below. These studies provide evidence that PTLD is an infection-associated condition with a distinct biology. Here and throughout, we adopt the terminology PTLD, as previously proposed (44, 118), to describe this condition.

The diagnosis and treatment of patients with PTLD face significant challenges. Objective tests to diagnose PTLD currently do not exist. Instead, diagnosis is made clinically through a process of documenting a prior history of LD and excluding other potential causes of persistent symptoms. Currently there are no prognostic indicators to stratify LD patients for their susceptibility to PTLD, but elevated levels of the T-cell chemokine CCL19, IL-23 or muted B-cell response were recently reported to be more common among PTLD patients compared to those that return to health following initial treatment (119–121). For patients that progress to PTLD, there are no FDA approved curative therapies. Safety and efficacy data for off-label treatment regimens and complementary therapies for PTLD are not well-established. Objective tests to determine whether a patient is cured or responsive to therapy also do not exist.

Objective laboratory tests that improve the diagnosis and treatment of this patient subgroup appear to be on the horizon in part due to investments in the creation of carefully constructed cohorts and biorepositories of well-defined patients with PTLD (see section Biorepositories and Research Cohorts). For example, the first biomarker of PTLD to be identified in one cohort that was then confirmed in a second, independent cohort of PTLD patients in the USA was published in 2020. This metabolomic signature discriminates clinically cured LD patients without PTLD from LD patients with PTLD (59). Alteration of gene expression was shown to persist after treatment for early LD among a small sample of patients followed longitudinally for 6 months. While no transcriptome signature was identified that could discriminate between patients that return to health after treatment compared to those that do not, the signature may lead to new objective tests for early LD (67). A gut microbiome signature was also recently identified that could distinguish PTLD patients from both healthy controls and ICU patients, further suggesting that PTLD is a distinct and definable disorder even if the underlying etiology remains uncertain (58). Other biomarkers related to LD and PTLD are reviewed below (section Pathogenesis). Taken together, these findings indicate that novel diagnostics and treatments for PTLD are now within reach.

## Fundamental Knowledge

### Genomic Insights From Borreliaceae Lineages

Between 1982 and 2010, the *B. burgdorferi* species complex, known as *B. burgdorferi sensu lato*, steadily expanded from 1 to 18 species (sometimes referred to as genospecies) as isolates from tick vectors, their hosts, and patient samples were characterized [reviewed in (122)]. A subset of these species are associated with human disease. *B. burgdorferi sensu stricto (Bb)* in the USA, as well as *B. afzelii* and *B. garinii* in Eurasia are the most common agents of LD in the Northern hemisphere. Cases of LD in Europe are also caused by *Bb and B. bavariensis* (123), but are less common. *B. spielmanii* (124), *B. bisettiae* (125–127), and *B. lusitaniae* (128, 129) have been identified in human specimens but their clinical importance is less clear. *B. valaisiana* has been identified in human specimens (130), but others have recently provided compelling reasons why existing evidence does not support it being considered a human pathogen (131). Additional species have been identified in tick vectors or their hosts, but not in patient samples.

The genus *Borrelia* was recently divided into two genera (132, 133). This division groups the tick- and louse-borne relapsing fever (RF) agents and their relatives into the genus *Borrelia* and the LD agents and their relatives into the genus *Borreliella*. Historically, *Borrelia* species were classified according to whether they were transmitted by hard-bodied or soft-bodied ticks (134). The advent of molecular characterization through genetic analyses, and more recently, whole genome sequencing, has led to a refinement in our ability to classify spirochetes that are morphologically very similar. The division of the genus is based on phylogenetic and comparative genomic analyses using 38 public genomes of 18 different species. Due to naming standards, RF agents retain the genus *Borrelia* since they were the first species identified in the genus. The reclassification of *Borrelia burgdorferi sensu lato* species to the genus *Borreliella* means that their names will change but the abbreviations are retained, e.g., *B. burgdorferi* for *Borreliella burgdorferi (Bb)*. The proposal generated considerable debate across the scientific community (135, 136). While adoption of the new naming convention has thus far been low in recently published literature, canonical bacterial reference manuals (133) and scientific databases have begun to incorporate the change.

New clinically relevant *Borreliella* species continue to be discovered [reviewed in (134, 137), reviewed in (138), reviewed in (139, 140)]. In 2016, a new pathogenic species, *B. mayonii*, was identified in the upper Midwest of the USA through PCR analysis of more than 100,000 human tissue specimens collected between 2003 and 2014. Specimens from a total of six patients were deemed positive for *B. mayonii*, all presenting after 2012 with signs and symptoms consistent with LD (141). The genomes of two *B. mayonii* isolates have been sequenced, with notable differences from *Bb* (strain B31) including the absence of the complement inhibitor Cszp and dozens of other proteins (142).

*Bb* has one of the most complex genomes of any bacteria characterized to-date [reviewed in (143)]. *Bb* contains a single linear chromosome of ~900 k base pairs (bp) with between 7 and 21 different plasmids, ranging in size from 5 to 84 kbp as reviewed from the genome sequences for 27 *Bb* isolates determined since the elucidation of the *Bb* B31 genome sequence in 1997 (144). The single chromosome appears to be very constant in gene content and organization across these *Bb* isolates. Overall, dozens of *Borreliella* isolates have been sequenced (144–147). These include the partial genomes of 64 isolates recently identified from collections across Canada, an emerging area for LD (148). To-date, chromosome assemblies have been reported from these isolates without, however, an equivalent analysis of the plasmid content. In the *Bb* genome, the conserved linear chromosome encodes most housekeeping genes, while the variable set of linear and circular plasmids encodes most of the outer membrane lipoproteins (149–151). Three plasmids, cp26, lp17, and lp54, are conserved across sequenced isolates of *Bb* to-date [reviewed in (143)]. The content and number of the plasmids however appear much more variable with some loss of these once *Bb* is *in vitro* cultured for an extended period of time. It is noteworthy that while extra-chromosomal DNAs are often referred to as plasmids—non-essential DNA that carries pathogenic and/or material that infer a selective advantage in a particular situation—some *Bb* “plasmids” carry essential genes and are more akin to mini-chromosomes. Knowledge about plasmids is currently limited due in part to the constraints next-generation sequencing technologies impose with short reads and the subsequent challenges with assembly. Long-read sequencing technology is poised to enhance our understanding of plasmid content and their dynamics across species and strains (150, 152).

Characterization of *Borreliella* isolates at the genome level is important to determine how genomic variation correlates with different disease phenotypes [reviewed in (122, 157)]. Molecular typing is used to classify distinct lineages of *Borreliella*. Common methods include pan-genome snp analysis (153), multi-locus sequence typing (MLST) (154), ribosomal RNA intergenic spacer typing (RST), and outer surface protein C (OspC) typing [(150), reviewed in (155)]. These methods for the classification of strains of *Bb* yield different numbers of distinct groups. Isolates of *Bb* from clinical samples collected from patients in the USA and Slovenia were recently compared and distinct differences were observed across genotypes, clinical manifestations, and inflammatory potential (156). In another recent study, serial blood samples obtained over a 21 day period from four patients with acute LD were assessed for infection with *Bb* via a novel direct detection method that combines PCR and electrospray ionization that is also able to discriminate different *Bb* genotypes. Two of 4 patients were infected with more than one genotype of *Bb*. Notably, the dominant *Bb* genotype changed over time in these two patients during antibiotic treatment (65). Infection with heterologous strains of *Bb* in humans is consistent with studies of pathogen burden in both ticks and vertebrate hosts (see section Transmission of *Bb* via Ixodes spp. Vectors). New technologies and high-throughput screening methods are advancing our understanding of which genes in the *Bb* genome are critical for ecologically or clinically relevant phenotypes, such as infectivity, tissue tropism (157) or drug tolerance. In a series of recent experiments, transposon insertion sequencing (Tn-seq) was used to identify genes associated with mammalian infection, resistance to oxidative stress, and survival in tick vectors (158–160).

LD agents are believed to inflict damage to people through inflammation caused by their immune response. No exotoxins have been identified in the genome with similarity to any previously described exotoxin found in other bacterial species, and the necessary secretory components appear to be absent. In addition, unlike most classical diderms, *Bb* does not produce the endotoxin Lipopolysaccharides (LPS) (144). A genome-wide proteome screen for immunogens in *Bb* (B31) using sera from patients with natural infections found that about 15% of the 1,292 open reading frames evaluated code for products that are immunogenic (161). More than 120 lipoproteins are encoded in the *Bb* genome (162) and represent nearly 8% of open reading frames (163). Of 125 lipoproteins examined experimentally, 86 of these are secreted to the bacterial surface (162).

### Proteomic Insights From Borreliaceae Lineages

The application of mass spectrometry-based proteomics has recently gained interest in the field of *Bb* and LD research to advance the understanding of disease pathogenesis and develop potential diagnostic methods and vaccines. Progress in *Bb* proteomics has been limited primarily to the proteins contained in the chromosome, while proteins encoded in the extra-chromosomal plasmids remain poorly characterized. In either case, attention has also been focused substantially on proteins encoded by genomic sequences of *Bb* that are homologs of B31—the vastly used isolate that was first collected from Shelter Island, New York (164). Further, very few laboratories working on proteomic workflows generate high-quality genomic data from different isolates of *Bb*. Nevertheless, advanced mass spectrometry-based proteomics provide unmatched information such as protein identification, quantification and post-translational modifications. The characterization of the *Bb* proteome enables basic science research and the development of vaccines and diagnostics (44, 165). Though several proteomic studies have been published, no high-resolution mass spectrometry (MS) data is represented in a publicly accessible repository for the community to access and utilize in research goals (162, 166, 167). The recent construction of the *Borreliella* PeptideAtlas repository (http://www.peptideatlas.org/) provides a unique community resource that contains large-scale assembly of observed and MS-derived validated data uniformly processed through the Trans-Proteomic Pipeline (TPP) (168). This database contains the proteome information from *Bb* isolates B31, 5A4, 297 and MM1 where 39,145 distinct peptides are validated and represent 1,283 *Bb* proteins. In addition to the unique peptide identification data, post-translational modifications are also presented with their validated MS spectra. The *Borreliella* PeptideAtlas is a dynamic proteome resource in terms of size and complexity and is continually updated as new datasets of *Bb* proteomes become available. These new datasets are ingested, processed through the TPP data analysis pipeline to ensure low false-discovery rates and presented in the conglomerated relational database for all users to explore and utilize. For example, the data can be mined to provide leading candidates across the isolates represented and the experimental conditions they were subjected to allowing exploration of how *Bb* adapts to, and is able to survive in a wide variety of environmental conditions. This resource provides a foundation for researchers to understand the dynamics of proteome organization throughout stages of infectivity and to generate targets to arrest infectivity.

### Transmission of *Bb via* Ixodes Spp. Vectors

The black-legged ticks, *Ixodes scapularis* and *Ixodes pacificus* on the West Coast, are the primary vectors of *Bb* in the USA. In endemic areas, the proportion of *Ixodes spp*. ticks infected with *Bb* can be remarkably high. One recent survey of the pathogen burden of 197 *Ixodes scapularis* ticks collected from New York and Connecticut where LD is endemic, revealed 111 (56%) ticks were infected with *Bb* and 37 (19%) were co-infected with more than one human pathogen (169). The high pathogen burden is consistent with previous tick surveys in the same region (170). In contrast, the percent of infected ticks in other regions of the USA is much lower. In recently published surveys across California, for example, fewer than 5% of *Ixodes* spp. ticks were infected (17, 170). *Ixodes* ticks sometimes carry multiple strains of *Bb* (65, 150, 171–173) that may impact the course of disease in people that are co-infected. One tick survey showed that 39% of *Ixodes* ticks in North America are infected with multiple genotypes of *Bb* (170).

*Ixodes scapularis* ticks feed only once per life stage (larvae, nymph, adult) after hatching from eggs. They primarily acquire *Bb* through feeding on infected vertebrates that are mostly mammals, but also some species of birds (174). *Bb* spirochetes colonize and persist in the tick until some are transmitted to a new host during the next feeding, while others remain in the tick during molting and the next life stage (175). *Bb* spirochetes are not transmitted from the adult tick to the egg, or it may occur only rarely. While transovarial transmission (TOT) of *Bb* in *Ixodes spp*. ticks has been reported in the literature, these reports may be attributable to confusion between *B. miyamotoi*, a relapsing fever agent, and *Bb* (176, 177).

*Bb* spirochetes have evolved multiple mechanisms to aid survival in adverse environments throughout the enzootic cycle. While colonizing the tick gut they must impede immune defenses of the tick, those of the host blood meal, and coexist with other residents of the tick gut microbiome. After their migration from the tick gut through the tick body cavity (hemocoel) and into the tick salivary glands, *Bb* spirochetes are expelled during feeding into the next host where they must again impede immune defenses and change their gene expression to survive and establish infection in a vertebrate host. The diverse and dynamic interactions between *Bb* and *Ixodes* ticks during *Bb* colonization, persistence, and transmission was recently reviewed elsewhere (178), but several recent findings are worth highlighting here.

The stability, abundance and diversity of the tick gut microbiome in *Ixodes* spp. is unsettled (179, 180), but there is evidence that the microbiome impacts the ability of *Bb* to effectively colonize the tick gut (181). The *Bb* genome lacks interbacterial defense pathways, which suggests they may not thrive in polymicrobial environments and the presence of certain taxa may interfere with colonization and persistence in the tick gut. For example, *Pseudomonas* possess genes for a Type VI secretion system that can deliver toxins to bacterial and eukaryotic members also residing in the tick gut microbiome (179, 182). Consistent with these findings, ticks infected with *Pseudomonas* are associated with lower burden of *Bb* (179, 182). Future research on the tick microbiome may lead to insights about approaches to disrupt pathogen colonization and transmission.

The tick salivary glands are important for transmission of *Bb* spirochetes. Tick saliva is essential to adequate tick feeding and contributes to human infection [reviewed in (183), reviewed in (184), reviewed in (185)]. During feeding, a tick alternates between secreting saliva and ingesting blood meal. Tick saliva not only carries pathogens from the tick into the host, but also contains molecules that function to aid the feeding process by creating blood flow through vasodilation and anticoagulative properties (186), blocking pain and itch (187, 188), impeding wound healing (189), and suppressing host immune response (190, 191). The composition of proteins expressed in tick saliva changes during feeding, which suggests another potential mechanism for host immune evasion through antigenic variation (192). A greater understanding of the components of tick saliva that support the infectivity of *Bb* may provide new routes to the prevention of LD.

### Pathogenesis

The pathogenesis of LD is believed to be driven in large part by the immune response of patients, although the underlying causes of ongoing inflammation and tissue damage in different stages of LD remain an active area of research. The microbial origin and inflammatory nature of untreated LD is better understood than the pathophysiology that underlies persistent signs and symptoms of disease experienced by some individuals following antimicrobial treatment. The dominant hypotheses about potential mechanisms underlying PTLD are immune inflammation and dysregulation and persistent infection and/or persistent antigenic debris. In this section, we review progress in the past 5 years on our understanding of pathophysiology of LD, starting with untreated LD. Then, we will review new evidence that has emerged related to persistent disease following treatment.

#### Immune Activation and Inflammation in Untreated LD

Inflammation is an important mechanism the body uses to aid in the elimination of pathogens. However, too much inflammation, such as what happens during a “cytokine storm” among some severe COVID-19 patients when cytokines are overproduced, can overwhelm the body and cause grave tissue injury. An aberrant inflammatory response is also what underlies autoimmune disease (193). In this section we review immune activation and inflammation associated with LD that begins soon after an infection with *Bb* from a tick bite.

The skin is an important first barrier to *Bb* infection. Interactions between the pathogen and human skin begin at the site of the tick bite where *Bb* spirochetes invade then disseminate outwardly, sometimes causing circular or elliptical EM lesions in their wake. One study induced suction blisters over EM lesions of LD patients in order to characterize the dermal leukocytes and cytokines of the aspirates from the skin. They found the aspirates to be enriched for T cells, monocytes/macrophages, and dendritic cells (DCs) compared to uninfected controls. Two cytokines, IL-6 and INF-γ, were predominant in the EM lesions (194).

The response of the innate immune system, including complement pathways and acute-phase proteins (APPs), occurs more or less immediately while the adaptive B- and T-cell response may take days to weeks. Macrophages and dendritic cells resident in the skin express a range of pattern recognition receptors (PRRs) that initiate signaling cascades and inflammatory responses as an early line of defense when they encounter and internalize a *Bb* spirochete [reviewed in (195)]. A yeast display screen of >1,000 extracellular and secreted human proteins identified direct interactions between *Bb* isolates and one human host factor, Peptidoglycan Recognition Protein 1 (PGLYRP1). *In vitro* assays show recombinant PGLYRP1 binds with purified peptidoglycan from *Bb* and have borrelicidal activity (196). In murine models deficient in PGLYRP1 (PGLYRP1^−/−^), pathogen burden of experimentally infected mice were higher than wild-type in hearts and joints but not skin. Moreover, PGLYRP1^−/−^ mice had reduced IgG serum levels and elevated proinflammatory cytokines (IFN-γ, CXCL9, CXCL10). The relationship between PGLYRP1 and pathophysiology of LD in humans is not yet known.

Phagocytic cells will attach to a spirochete via a repertoire of host cell surface receptors. These include Complement Receptor (CR) 3 and CD14 (197–200), urokinase receptors (uPAR) (200, 201), scavenger receptors such as macrophage receptor with collagenous structure (MARCO) (200, 202), some Toll-like receptors (TLRs), C-lectins, sialic acid-binding immunoglobulin-like receptors (siglecs), Fc receptors, or others (200). The attachment of *Bb* to host cell surface receptors leads to signaling that induces innate and specific adaptive immune responses, as well as clearance of the pathogen through phagocytosis. For example, the host cell surface receptor, Toll-like receptor 2 (TLR2), is a PRR that binds to ligands on the *Bb* cell surface that display certain pathogen associated molecular patterns (PAMPs). When a PRR senses a pathogenic ligand, it upregulates an inflammatory response, which commonly includes the induction of pro-inflammatory cytokines, chemokines, and antimicrobial peptides (AMPs); however, different types of activated PRRs induce different gene expression patterns. The production of pro-inflammatory cytokines, such as interleukin-6 (IL-6), recruits blood cells to the site of inflammation and induces the production of APPs, such as C-reactive protein (CRP).

The internalization and degradation of engulfed *Bb* spirochetes is sensed by intracellular receptors, including the NOD-like receptors (NLRs) and endosomal TLRs. These receptors activate signaling pathways that induce the production of cytokines, including interferons (IFNs). These are classified into three main types: I (IFN-α or IFN-β), II (IFN-γ), and III (IFN-λ) [(203), reviewed in (204), reviewed in (205)]. Type I IFNs are induced by *Bb* DNA or RNA through TLR7 and TLR9 (203), as well as TLR8 in monocytes (206). Type II IFN is produced by innate lymphocytes, including natural killer cells (NK cells) and by T helper cells (Th) (207). *In vitro*, type III IFN is induced in peripheral blood mononuclear cells (PBMCs) through TLR7 by live spirochetes or purified *Bb* RNA (208), perhaps controlled by *Bb* plasmid lp36 (203). The role of the type III IFN pathway in disease pathogenesis has not been fully elucidated, although clinical isolates associated with disseminated disease produce stronger IFN responses (Type I and III) (203).

The early stages of LD show an elevation of inflammatory markers and immune mediators (69, 119, 209, 210). CRP is an APP that is used clinically as a marker of inflammation that may denote infection, malignancy or cell stress (211). Within a few hours of an infection or other stressor, cytokines secreted by immune cells will enter the bloodstream and cause the liver to secrete CRP. Generally, the normal range of CRP for healthy adults is <10 mg/L; moderate elevation is 10–100 mg/L; and marked elevation is >100 mg/L (212). A longitudinal assessment of CRP levels in serum samples from 44 LD patients presenting with one or more EM lesions and followed for 2 years after treatment, showed a significant elevation of CRP prior to treatment and a rapid decline to control levels following treatment (209). Elevated levels of CRP at the pre-treatment visit were seen again in a larger study by the same group, along with 6 additional elevated markers, including CCL19, ferritin, fibrinogen, IFN-γ-induced protein 10 (IP-10), monokine induced by IFN-γ (MIG), and serum amyloid A (SAA) (119). The first systematic study of APP levels in serum samples from patients at different stages of LD and healthy controls was recently conducted (210). Consistent with the previous studies, CRP levels were most elevated in patients with early localized (single EM, *n* = 18) and early disseminated (multiple EMs, *n* = 17) LD, with 33 and 71% of patients, respectively, having CRP levels >10 mg/L (210). More recently, a proteomic analysis of serum proteins in 70 LD patients with one or more EM lesions at the time of diagnosis, identified six proteins, including CRP, with significantly altered serum levels shared across two independent cohorts (69). The other five elevated proteins are APOA4, C9, CST6, PGLYRP2, and S100A9. Two independent studies have also shown that the chemokines, CXCL-9 and CXCL-10, both known to be associated with IFN-γ production and thus a “TH-1 type” of immune response, are elevated at time of diagnosis in EM+ LD patients in the USA and Europe (120, 209). Among a European cohort, patients with symptoms at study entry had significantly higher levels of CXCL9 compared to patients without symptoms (120).

#### Inflammation and Immune Dysregulation Among Patients With Persistent LD

The immune profiles of LD patients with persistent health problems following antibiotic treatment are not consistent across well-characterized subgroups, however several potentially important immune mediators have emerged within subgroups. Next, we will review each of these.

##### CRP in Patients With Persistent Symptoms Following Treatment

The levels of CRP in other stages of LD (subsequent to early localized or early disseminated, as described above) and patients with persistent signs or symptoms following treatment are less consistent. Among patients with antibiotic-refractory LA (*n* = 11), 55% have serum CRP levels >10 mg/L while patients with early neurologic, late neurologic, and antibiotic responsive LA showed no significant difference in CRP levels compared to controls. For patients with PTLD (*n* = 74), 15% had CRP levels >10 mg/L compared to 4% of patients with LD that returned to health following treatment (*n* = 68) (210). For some diseases, such as cardiovascular disease, serum CRP in the range of 3–10 mg/L is considered of clinical value for understanding an underlying inflammatory process or stratifying risk in some patients (213–215). A significant proportion of both, antibiotic-refractory Lyme arthritis (73%, *n* = 11) and PTLD (55%, *n* = 74) patients have serum CRP levels in this range (210). While these data are suggestive of an ongoing inflammatory process in these two patient subgroups, more research is needed to better understand the role of CRP, along with other markers of inflammation, and the underlying mechanisms at work in affected patients.

##### IFN-γ and Antibiotic-Refractory LA Patients

Antibiotic-refractory LA patients without evidence of an ongoing infection, show excessive IFN-γ production. So far, this biomarker appears to be specific for LA as serum levels of IFN-γ are not significantly elevated among EM+ LD patients at the time of presentation compared to controls, nor are levels of this cytokine significantly elevated in patients with PTLD (119). A recent study did a comparative analysis of synovial tissue from patients with LA, rheumatoid arthritis, and low inflammation osteoarthritis and found that LA is unique, showing elevated levels of IFN-γ and IFNγ-producing T cells and NK cells in synovial fluid (216). This study also identified and characterized a population of fibroblast-like synoviocytes (FLS) that are hypothesized to be involved in mediating persistent inflammation. These cells, when activated by IFN-γ *ex vivo*, expressed genes and pathways that overlapped with that seen in postinfectious LA synovial tissue (216). This suggests that the FLS are driving ongoing inflammation and suppressed wound healing in an IFNγ-dependent manner.

##### CCL19 Among PTLD Patients

The T-cell chemokine, CCL19, was associated with susceptibility to PTLD. In a recent study, sixty-four cytokines, chemokines, and inflammatory markers were measured in serum collected from 76 EM+ LD patients at six visits over 1 year. Eleven patients (14.5%) went on to develop persistent symptoms that impacted daily functioning following treatment and were classified as having PTLD. Twenty-nine patients (38.2%) had symptoms following treatment without a functional impact on daily living and 36 patients (47.37%) returned to health. Patients with CCL19 above 111.67 pg/ml at the (1-month) visit were 12.6 times more likely to meet criteria for PTLD at the 6 and 12 months timepoints (119). In a murine model, CCL19 along with IL-23, were associated with a pathological TH17 response in experimental autoimmune encephalomyelitis (EAE) (217). However, it has not been fully assessed to which extent the TH17 pathway is induced in LD patients.

##### IL-23 Among European PTLD Patients

A cytokine, IL-23, associated with IL-17 production and thus “TH-17”-type responses, is elevated in early LD and remains so among many European patients that have persistent symptoms following treatment. Eighty-six EM+, untreated LD patients that enrolled in an antimicrobial drug trial in Europe and were followed longitudinally for a year after treatment and were assayed for 26 cytokines and chemokines at 4 time points. Among the patients studied, 45 had symptoms following treatment consistent with having PTLD. One cytokine (IL-23) and two chemokines (CXCL9 and, to a lesser extent, CXCL10) showed significant differences across groups. Most patients with detectable IL-23 levels at study entry went on to develop PTLD and IL-23 levels remained elevated in these patients with ongoing symptoms. The seven patients with the highest levels of IL-23 (≥230 ng/mL) all went on to develop PTLD. Thus, an aberrant TH17-related immune response might be contributing to symptoms in patients with elevated IL-23 (120). It is interesting to note that no significant difference was identified in serum levels of IL-23 among the longitudinal cohort of LD patients from the USA, while CCL19 was not noted to be significantly elevated in the European cohort (119, 120). One potential reason for these differences is the difference in the primary agents of LD in Europe and the USA: *B. afzelii* and *B. garinii* vs. *Bb*. However, the mechanisms underlying these and other differences between LD in North America and Europe remain poorly understood.

##### Autoantigens and Self-Reactivity in LA Patients

The origin of autoimmune disease (AD) in humans have previously been thought of as triggered by microbial infection, which may serve as a catalyst for development of responses to self-antigens [reviewed in (218)]. Several autoimmune diseases are associated with bacterial triggers, such as gastric autoimmunity and Guillain-Barré syndrome triggered by infection with *H. pylori* and *C. jejuni*, respectively [reviewed in (219)]. One proposed mechanism for possible LD-associated autoimmunity is the circumstance where sequence or structural homology between human and *Bb* proteins cause B- and T-cell receptors to cross-react with an epitope on a *Bb* protein (the intended target) and a human protein (the unintended target). This could lead to ongoing inflammation of tissue and has been most well-studied in patients with LA (220–228). Four self-proteins (autoantigens) recognized by CD4 T cells have recently been identified in LD patients with LA through the use of tandem mass spectrometry on peptides in complex with MHC class II receptors (HLA-DR) (229). The four autoantigens identified include peptides from endothelial cell growth factor (ECGF), apolipoprotein B-100 (apoB-100), matrix metalloproteinase-10 (MMP-10), and annexin A2 proteins. Autoreactivity to these self-proteins appear to be primarily associated with LD, except annexin A2, which was associated with other rheumatic diseases (228), and potentially, to severe COVID-19 (230). To-date, no specific autoantigens have been identified reliably in LD. For the autoantigens that have been identified, we currently do not know whether the observed autoreactivity is induced by the presence of specific *Bb* antigens.

#### Persistence

##### Persistent Antigenic Debris

Recent studies of *Bb* peptidoglycan have renewed interest in the potential roles this immunogenic macromolecule may play in LA and, more broadly, in disease pathogenesis. Peptidoglycan (PG) is an essential biopolymer that acts like a molecular bag—surrounding the bacterial cytoplasm and preventing cell bursting due to osmotic pressure. The cell walls of virtually all bacteria contain PG, and chemical and structural conservation is apparent [reviewed in (231)]. Glycan strands, made up of the repeating disaccharide *N*-acetyl-glucosamine and *N-*acetylmuramic acid, are cross-linked by peptides composed of often alternating L and D-amino acids. Deviations from this chemical and conformational arrangement are rare, which make PG a quintessential pathogen-associated molecular pattern (PAMP) [reviewed in (232, 233)]. Recognition of bacterial peptidoglycans by innate immune system receptors [e.g., Toll-like receptors (TLRs), PG recognition proteins (PGRPs), and NOD proteins] leads to inflammation and the production of cytokines that can result in host tissue damage. Immune response(s) to bacterial PGs have been associated with symptoms of infections such as gonorrhea (234, 235), chronic gastritis, and pertussis (236). There has been some evidence for a potential role for peptidoglycan in several autoimmune diseases, including rheumatoid arthritis (237) and multiple sclerosis (238, 239).

The cell envelope of *Bb* contains a peptidoglycan (PG^Bb^) that so far appears to be chemically unique, but conserved amongst some spirochetes. For example, close *Borreliae* relatives in the Relapsing Fever clade and *Treponema* genus have been reported to possess L-Orn-type PG (240, 241). For bacteria to grow, divide, and ultimately cause disease, PG is continuously remodeled—small muropeptide fragments are removed and replaced with multimers. Unlike many other bacteria, *Bb* lacks the genetic components necessary for recycling the excised muropeptide fragments back into the cytoplasm. Instead, muropeptides are shed during growth and accumulate in logarithmic fashion that correlates with spirochete density (102). Analysis of radio-labeled, PG-associated amino acid over time, indicates that *Bb* sheds ~45% of its PG per generation (102). Despite the overall abundance PG shedding would cause, its role in LD pathogenesis remains to be fully elucidated.

PG^Bb^ elicits an immune response in humans. This was first shown in 1990 when PG isolated from *Bb* was injected subcutaneously into the forearm of a volunteer, one of the co-authors of the report, who then experienced intense inflammation at the injection site for 72 h (242). Jutras et al. detected PG^Bb^ in 94% of synovial fluid samples collected from 34 patients with LA (102). They also demonstrated the connection between PG^Bb^ and disease pathogenesis through tail vein injection of PG^Bb^ into mice and the subsequent observation of acute arthritis (102).

Much remains to be determined about the role of PG^Bb^ in LA and, more broadly, in LD. For instance, are the PG remnants that of dead/dying bacteria following phagocytosis or antibiotic therapy or are they shed muropeptides? Transcript levels of Lysozyme—the human enzyme responsible for degrading PG—are elevated in LA patient SF (216), so why isn't it working or is the substrate absent (polymeric PG vs. muropeptides). The fate of shed muropeptide fragments, or their dwell time in different anatomical sites, is not known. In fact, the exact chemical composition of released muropeptide(s) is yet to be determined. Since released muropeptides must contain L-Orn (102), one intriguing possibility is that PG chemistry affects the response and/or half-life in the human host. Do germline variants or differential expression of human PGLYRP1 impact the pathogenesis of LD? Clearly, much remains to be elucidated, but methods toward preventing the human responses to PG^Bb^ or eliminating the lingering antigen entirely, are two attractive avenues of future therapy in patients with persistent LD.

##### Persistent Infection

Like most bacteria, *Bb* are able to change their cellular phenotype in order to better survive in adverse conditions. *Bb* form persister cells, *in vitro* (85, 243) or when exposed to antibiotics [(243), bacterial “persistence' reviewed in (244), reviewed in (245, 246)]. The connection between persister cells, atypical morphological forms of *Bb* and disease pathogenesis in LD remains poorly understood [reviewed in (247)].

*Antibiotic resistance vs. antibiotic tolerance*. Generally speaking, the ability of bacteria to grow in the presence of an antibiotic indicates resistance of a specific nature. Whether the antibiotic targets bacterial protein or nucleic acid synthesis, cell wall synthesis or integrity, or a specific metabolic pathway, uninhibited growth in the presence of that antibiotic demonstrates that the bacteria have acquired the ability to counteract that drug. Resistance may be inherent in the genome, acquired by horizontal gene transfer, or produced by mutation. *Pseudomonas aeruginosa*, for example, possesses multiple operons that encode efflux pumps (248) and *Mycobacterium tuberculosis* has become resistant by virtue of mutations which, for example, prevent the inhibitors of cell wall synthesis from binding to their target enzyme (249, 250). Alternatively, bacteria may stunt their own replication in the presence of a bacteriostatic antibiotic, thus minimizing the effect. The latter is non-specific and can be referred to as antibiotic tolerance. *Bb* encodes an efflux pump system, but specific resistance to antibiotics has not been clearly demonstrated (251). The generation of slow-growing or non-growing “persister” cells *in vitro* is a well-established observation for multiple bacterial species (252, 253). *Bb*, in particular, has been shown to tolerate multiple antibiotics (85, 243) and form persisters by a stochastic mechanism leading to a slow-growing phenotype (254). Persister cells *in vivo* have not been demonstrated. In natural infection, the ability of bacteria to establish dormancy is perhaps best exemplified by *Mycobacterium tuberculosis*. Such infections can be latent for years and often never result in fulminant disease; the entry into dormancy is likely influenced by hypoxia or other environmental stressors (255). Another pathogenic spirochete, *Treponema pallidum*, can enter a chronic dormant phase within the human host and reactivate as tertiary syphilis years after the primary infection (256). How or where *T. pallidum* persists is not known.

If entry into a dormant phase occurs *in vivo*, the possibility for bacteria to tolerate growth-inhibiting compounds is a legitimate possibility. *B. burgdorferi* may enter dormancy during long periods of nutrient deprivation within the tick or following treatment of a mammalian host with a bacteriostatic antibiotic such as doxycycline. The stress response of the bacteria to nutrient deprivation has been described (257) and this capability to survive harsh environments may well contribute to antibiotic tolerance as well (258).

*Bb infection, in particular*. The question of the effectiveness of antibiotic treatment for LD has been contentious among physicians and researchers for some time (91, 259–261). Among the challenges to determining if antimicrobial therapy is curative is the absence of reliable measures to determine that infection has been cleared from LD patients and the vague, non-specific symptoms with which patients present in PTLD (30, 106). The notion that spirochetes may persist in humans derives primarily from the proportion of patients who experience symptoms post-treatment (107, 262). A few studies have examined this phenomenon in humans—two in the U. S. [reviewed in (263)] and one in the Netherlands (264). For both studies in the USA, only patients that had been treated for acute (early) LD were included and the authors concluded that resolution of symptoms did not occur with a subsequent 90-day treatment with antibiotics (265). While the Netherlands study also showed no significant difference between longer term treatment of patients with chronic symptoms and short-term therapy, the inclusion criteria were less stringent, potentially allowing patients without LD into the study. More recently, spirochetes were detected in post-mortem brain samples collected from a patient who previously was diagnosed and treated for LD and subsequently experienced chronic symptoms, including dementia (266).

*Studies of persistence*. To date, multiple studies in animals have shown that *Bb* spirochetes do persist following antibiotic treatment of a disseminated infection (267–269). Even in humans, rare evidence of possible persistence was gleaned from feeding uninfected ticks on a patient with PTLD (270). In this study, only *Bb* DNA was detected while attempts to visualize or culture viable spirochetes from the xenodiagnostic ticks were unsuccessful. As others have noted, without the recovery of metabolically active spirochetes, this experiment is suggestive but is not a clear demonstration of persistent infection in humans (271).

Evidence from experiments performed in mice and dogs reveals that spirochetes can persist in the mammalian host after the administration of antimicrobial drugs. In one study, dogs were treated with a 30-day course of amoxicillin or doxycycline 2 months after infection (267). Spirochetes were recovered from tissues in 3 of 12 dogs. Skin punch biopsy samples from nearly all dogs were PCR-positive for *Bb* after treatment. Interestingly, serum antibodies to *Bb* declined post-treatment, but after the dogs were kept 6 months in pathogen-free housing, their antibody titers rose, indicating recrudescence. Spirochetal persistence has been examined in mice using xenodiagnostic studies in which naïve ticks were placed on infected mice that received a course of antibiotics (272–274). Those ticks acquired spirochetes from the mice post-treatment, detected by fluorescent imaging (272) or PCR (273, 274). Two of these studies examined the existence of persistent spirochetes as a function of the time elapsed prior to treatment (273, 274). Spirochetal DNA was more frequently detected in xenodiagnostic ticks (XT) that fed upon mice treated 4 months post-infection than from those treated 3 weeks post-infection. When XT that had acquired organisms from antibiotic-treated mice fed upon naïve mice, the mice harbored spirochetal DNA in multiple tissues (detected by PCR), but organisms could not be recovered by tissue culture. Finally, studies in primates have shown that morphologically intact spirochetes can persist following antibiotic treatment (268). In a subsequent study, not only were the spirochetes found intact after treatment, but were shown to be transcriptionally active and were detected in multiple tissues, including the brain and heart (275, 276). This is evidence to suggest that the organisms are not fully cleared and may be attenuated for infection or for recovery by tissue culture.

With rare exception (127), only *Bb* genetic material (DNA or RNA), antigen, or non-culturable spirochetes have been detected following antibiotic treatment of an established infection. In none of the aforementioned animal studies has the pathogen been recovered as indicated by spirochete replication in culture soon (1–2 weeks) after inoculation of the standard BSK medium with tissue or tick specimen. Some experts in the field have therefore surmised that the spirochetes are non-viable and therefore that the infection is not persistent (103, 271, 277, 278). Evidence of resurgence in mice that were evaluated a year after antibiotic treatment contradicts this notion of non-viability (269). In that study, the amount of *Bb* genetic material in each mouse was quantified and found to increase from a very low level a few months after treatment to levels as high as in untreated animals at 12 months after treatment, indicating that the spirochetes replicated. The *Bb* bacteria present after antibiotic treatment, which are metabolically active and appear to be capable of resurging *in vivo* or resuscitated under the right culture conditions have been deemed “viable, but non-culturable (VBNC).”

The VBNC state is not unique to *Bb*, but rather, it is known to occur in over 100 other bacterial species studied to date (279). Entering dormancy of this “nonculturable” type is very common for bacterial pathogens (280, 281). The VBNC state has been characterized as a deeper state of dormancy than that of persister cells, observed in several *Vibrio* species, *E. coli, Campylobacter, Burkholderia, Listeria, Salmonella*, and *Helicobacter* (281–283).

Persistent infection with *Bb* is difficult to rule-in or out as an explanation for LD patients with ongoing symptoms due to the challenge of culturing viable spirochetes from human specimens except in the earliest stages of infection, prior to antibiotic treatment (21). The failure to reliably isolate metabolically active spirochetes from patients does not exclude the possibility that they exist in some patients with ongoing health problems.

## Prevention

### Ecological Prevention

In the USA, *Bb* is the most common vector-borne pathogen; LD comprises 62.6% of all vector-borne diseases and 81.2% of all tick-borne diseases (1). There is an increasing trend of new cases in counties and states neighboring high-incidence regions, indicating a spread of the pathogen and disease risk in new geographical areas (52). The current complexities around the diagnosis and treatment of LD and PTLD suggest a growing need for primary prevention and to understand the intricacies of the ecological factors that impact disease risk.

LD risk, and the broader goal of prevention of LD, is commonly viewed through two lenses: an ecological approach that focuses on characteristics of the tick vector, its hosts and the pathogens it transmits; and a human behavior approach that examines how behaviors and attitudes of human individuals, such as frequency of outdoor activities or use of protective equipment, change risk to disease (284, 285). Indirect factors regarding host populations, abiotic conditions, and land use or land coverage have also been found to increase disease risk, yet the magnitude of impact or relation to tick encounters and infection risk is still not fully understood (284, 286).

Popular ecological preventative techniques gravitate around reduction of host populations, reduction of ticks, and reduction of pathogen infection in ticks or hosts. Popular human-behavioral strategies include altering the risk of exposure of humans through behavioral changes associated with self-protection, use of outdoor space, and modifications of the environment. White-footed mice are the primary reservoir for *Bb* and their density has been shown to affect LD risk (287–290). The culling of white-tailed deer is a common preventative technique, but research sheds doubt on the viability or practicality of mass culling, suggesting that the technique is only effective on islands or closed populations where complete elimination can be accomplished (291). Personal protective measures, including checking body for ticks and use of tick repellent are frequently promoted by government and public health agencies. Some of these measures have been shown to reduce disease risk, yet effectiveness may be as low as 20–40%, with some practices like checking one's body for ticks being found ineffective (284, 292, 293). One challenge to prevention is the fact that nymphal ticks are as small as a poppy seed, and their bites can easily go unnoticed. Land usage or cover has shown strong trends of being impactful on exposure to LD, but still being researched is the spatial scale of land usage (284). Questions of land use in residential spaces or neighborhoods are still being explored, as well as the human movement within those spaces (294).

Several research projects have been initiated to further explore LD prevention by minimizing infection risk. The Tick Project is undertaking an immense randomized control trial of effectiveness of *Met52* fungal spray and Tick Control System rodent bait boxes for LD prevention across 24 neighborhoods in Dutchess County, New York, while also collecting and assessing data on the entomological and host population risk factors, tracking tick encounters, and documenting cases of LD and PTLD across these neighborhoods (295). New models are being created to predict the first incidences of LD in counties without any reported cases of LD based on abiotic and human behavior factors (296). Prediction models will need to account for climate change as a contributing factor to the expanding range of LD. In addition, there are still several gaps in our knowledge about effective preventative techniques that should be further studied: abiotic factors and the capability of predictive modeling, diversity of *Bb* strains in tick and host populations and its impact on disease risk, predator communities and their role on host communities, changing landscapes and urban spaces, and the costs, sustainability, and acceptability to the public of preventative techniques (286, 288, 290, 297).

### Human Vaccine

There is an urgent need for a safe and effective human vaccine that targets multiple pathogenic *Borreliella* species and strains, or even more broadly, common co-infections. Currently, there is no human vaccine for LD commercially available in the USA. In 1998, the FDA licensed Lymerix, a recombinant Osp-A based vaccine for the prevention of LD in adults. The vaccine required three shots over two tick seasons and was reported to be 76% effective in the prevention of LD after the third shot (49). The mechanism of action of this and other Osp-A based vaccines is antibody-mediated blocking of the transmission of *Borreliella* spirochetes from infected ticks while feeding on a human host (298, 299). Public demand and acceptance of the vaccine was low for a variety of factors reviewed elsewhere (300–304) and Lymerix was pulled from the market by its manufacturer, Smith-Kline Beecham in 2002. Interest from industry waned and other efforts to develop Lyme vaccine candidates were also abandoned, including those by MedImmune, Baxter and Connaught Laboratories (300).

Twenty years after Lymerix, there are now multiple efforts underway to develop next-generation human vaccines for the prevention of LD. One challenge that these vaccines must address is the genetic diversity of pathogenic *Borreliella* species and strains across different geographies. Even a single infected tick may carry multiple heterologous strains of Bb. Therefore, current vaccine candidates incorporate multiple immunogenic antigens or multiple serotypes of a single immunogenic antigen. Outer surface proteins, especially OspA-C, are the most common antigens selected among current human vaccine candidates, but other antigens and vaccine strategies are being studied [reviewed in (305)].

VLA15 is currently the only Lyme vaccine candidate in human trials. VLA15 uses recombinant outer surface protein A (OspA) from six different OspA serotypes of pathogenic *Borreliella* species, including *Bb* (OspA serotype 1), *B. afzelii* (OspA serotype 2), *B. garinii* (OspA serotypes 3, 5, 6), and *B. bavariensis* (OspA serotype 4) (306–308). In a similar approach, a prototype vaccine that uses bacterial ferritin nanoparticles fused with seven serotypes of OspA molecules from *Bb, B. afzelii, B. bavariensis* and *B. garinii* recently showed durable high-titer antibody response in both mouse and rhesus macaque animal models (309). In both vaccine candidates, the antigenic residues in Osp-A serotype 1 that were previously suspected (221) but never shown to be cross-reactive were removed (304, 309).

There are several promising strategies for the development of other novel human vaccines for LD in experimental and preclinical stages. Marconi et al. has conducted studies in dogs on a subunit vaccine that includes OspA and at least 14 immunogenic linear epitopes (“chimeritope”) from diverse isotypes of OspC. These outer surface proteins are expressed while *Bb* is in the tick gut (OspA) or during early human infection (OspC) [(305, 310–312), reviewed in (313)]. The OspC chimeritope is a component of the most widely used Lyme vaccine for dogs (314). By combining antigens that are expressed by *Borreliella* at different stages of infection, this vaccine has the potential to protect against spirochetes that are not blocked or killed while in the tick gut. This vaccine has not yet entered human trials. Another experimental vaccine targets Cspz, an outer surface protein involved in complement evasion (315).

The development of an anti-tick vaccine is one potential approach to protect people from multiple tick-borne diseases, including LD, as recently reviewed elsewhere (316). *Ixodes scapularis* ticks transmit 16 human pathogens (317) associated with tick-borne disease in the USA, including *Bb, Borrelia miyamotoi* (318, 319), *Babesia microti* (320, 321), *Anaplasma phagocytophilum* (320, 322), *Ehrlichia muris-like agent* (EMLA) (323) and Powassan virus (324, 325). During transmission to a human, bacteria interact with tick proteins in the gut and salivary glands. These interactions can influence whether transmission occurs. Increased protection might be conferred if any of several steps in the transmission cycle are inhibited by targeting one or several of these tick proteins simultaneously. For example, mice that were given antiserum to the tick protein, Salp15, and then were challenged with *Bb*, showed protection from colonization (326). Tick proteins may also elicit “tick immunity,” a process during which a host becomes resistant to tick bites because the ticks cannot feed properly (327). If a vaccine can be developed that creates tick immunity in humans, this may enable the prevention of LD, and other tick-borne diseases, especially for those that migrate slowly from tick to human (305, 328–332). Viruses, such as Powassan, can be transmitted from tick to the human host in mere minutes. The attachment time required for tick-borne pathogens to migrate from tick to host was recently reviewed elsewhere (333). Encouragingly, there is already one commercially available tick vaccine used for the protection of livestock against tick infestation, though not including *Ixodes spp*. ticks (334–336).

## Field Building

To improve our ability to better address Lyme and other tick-borne diseases, we need to attract researchers to the field and build shared resources that accelerate research progress such as biorepositories, genomic resources, animal models, and preclinical services (57). Interest in LD research over the past 5 years has remained relatively flat: the term “Lyme disease” is mentioned in around ~1,000 publications per year combined from PubMed Central in the USA and Europe. In this section, we describe several key resources for investigators seeking to study LD.

### Biorepositories and Research Cohorts

Well-characterized samples are an essential tool to help researchers develop and validate new diagnostic tests and to better understand the complexities of LD. Well-characterized sample sets can benefit medical providers, test developers, and the public at risk for LD (337). It is critical that sample users understand the criteria used to enroll participants, how samples were collected and stored, and what additional clinical and testing data may be available. Additional benefits can be realized when multiple sample users (test developers and researchers) are using the same well-characterized sample sets. Current sample sets available for researchers include the CDC Lyme Serum Repository (LSR) (337), the Lyme Disease Biobank (23), and samples from the Studies of Lyme Immunology and Clinical Events (SLICE) at Johns Hopkins University School of Medicine. Additionally, some investigators also have their own sample collections with, in some cases, blood samples, skin biopsy specimens and synovial fluid which form the basis for collaborative studies (69, 102, 338).

#### Lyme Disease Biobank

The Lyme Disease Biobank is a collection of more than 900 human biological samples that facilitates research in the field of LD and other tick-borne infections (TBI). Whole blood, serum and urine samples are collected from individuals presenting with the signs and symptoms of early LD with or without an EM lesion, individuals with later stages of LD including persistent LD, and unaffected individuals (endemic controls). Samples have been collected from the East Coast, Upper Midwest, and California. Robust clinical information accompanies the samples, including information about symptoms, EM (if present), current medications, history of LD and other TBI, medical history, and demographics. Photographs of EM lesions are also taken (if present). Participants enrolled with early LD also have the option of providing a convalescent sample 2–3 months after the initial blood draw. PCR testing is performed, in a blinded fashion, to confirm the presence of *Bb, Anaplasma phagocytophilum, Babesia microti*, and *B. miyamotoi* in the samples. Serologic assays for standard two-tiered testing analysis (ELISA followed by IgM/ IgG Western immunoblotting) have also been performed. Each participant's donation provides samples for ~50 research projects, with aliquots of whole blood (1 and 2 ml), serum (250 μl), and urine (1 ml) available to sample users in academia and industry. More than 10,000 aliquots have been distributed across 50+ diagnostic research projects. The characterization of samples from 550 participants enrolled on the East Coast and Upper Midwest is detailed here (23). Specific panels are also available, including an unblinded panel for projects earlier in development, panels of later stages of LD including Lyme carditis and neuroborreliosis, panels of other TBI, as well as samples from patients with persistent LD. A tissue bank was recently launched for post-mortem and surgical samples. Tissue donors have the option to link their MyLymeData registry profile to their tissue sample (339, 340).

#### Lyme Disease Research Center

The primary focus of the Johns Hopkins Lyme Disease Research Center (LDRC) is clinical translational research to advance the fundamental understanding of LD through the characterization of carefully constructed cohorts of LD patients and controls, as well as a clinical biorepository of blood and tissue biospecimens. The LDRC enrolls participants from an expanded Mid-Atlantic region into a variety of research protocols, which all collect detailed health histories, clinical, and behavioral data. Over 350 participants have been enrolled in ongoing longitudinal cohort studies (some followed for up to 10 years), which include patients meeting CDC criteria for early and late LD, as well as uninfected controls without LD. An additional 275 participants meeting the IDSA definition for PTLD have also been enrolled in a clinical case series study (44). The SLICE studies obtain a number of different biosamples including: a skin biopsy (in patients with acute LD), whole blood, serum, plasma, PBMCs, DNA, RNA, skin and fecal swabs, and most recently, urine. All these samples are processed in the laboratory, aliquoted, archived and stored. To-date, they have shared ~6,000 sample aliquots for collaborative initiatives. The center collaborates with key investigators who utilize these samples for immune profiling (209), transcriptomics (67, 68), proteomics (69), metabolomic (75), and microbiome-based studies (58). These studies generate rich and deep clinical and molecular data sets that may allow for new insights into LD pathophysiology, lead to new diagnostic and therapeutic approaches, and have contributed to the characterization of LD and PTLD longitudinally across dozens of clinical and neurobehavioral variables. The LDRC has demonstrated that PTLD is a definable condition that is distinguishable from those that “return to health” following infection and treatment for LD. With the ability to compare PTLD patients with controls uninfected with LD, the SLICE studies show that the rates of individuals with both symptoms and a decline in health-related quality of life are significantly higher in patients previously treated for acute LD than in controls (14 vs. 4%) (unpublished data). The growth of these cohorts over the years is also evidence that it is possible to not only recruit patients with PTLD into research, but also maintain their participation at high levels.

#### Long Island Outdoor Worker Cohort

To investigate the seasonal incidence and seroprevalence rates, a team of investigators at the Stony Brook School of Medicine assessed outdoor workers in the Hispanic/Latino immigrant population residing in Eastern Long Island and compared rates to those of non-immigrant outdoor workers. To further investigate occupational risk, they looked at differences of incidence rates in field workers and non-field workers within the Hispanic/Latino immigrant population. The study shows a significantly higher rate of *Bb* exposure among Hispanic/Latino immigrant field workers compared to those belonging to other occupations and in non-immigrant outdoor workers and also sheds light on the epidemiology, seroprevalence, and seasonal incidence of Lyme and other tick-borne diseases, as well as their clinical manifestations. Treatment and prevention of LD in this population can be especially difficult to obtain when multiple barriers are in place, such as poor health literacy, lack of preventative measures and limited access to healthcare in those with more risk. These findings underscore the necessity of improved education and preventative measures to better protect this vulnerable population (341, 342).

### Data Repositories

#### LymeMIND Commons

The LymeMIND center at the Icahn School of Medicine at Mount Sinai is developing LymeMIND Commons (https://commons.lymemind.org/), an online database and a search engine that contains collected data and metadata integrated from the consortium and other LD resources. LymeMIND Commons enables researchers with the ability to find and analyze various types of data and metadata related to LD. Beyond transcriptomics, data includes protein arrays, methylation profiling by high throughput sequencing, genotyping, and other platforms. The metadata in LymeMIND Commons is JSON serialized and hosted using the Signature Commons platform. The metadata within LymeMIND Commons is linked to external ontologies and other resources for each study. In summary, LymeMIND Commons serves as a unique resource to advance LD research.

## Discussion

More research into the prevention, diagnosis and treatment of LD is needed in order to address the significant health risks posed by this tick-borne disease. The annual NIH and CDC investment in Lyme and tick-borne diseases research has been relatively unchanged for decades and is small compared to many other infectious diseases. In 2015, the Steven & Alexandra Cohen Foundation established a research consortium involving over 30 leading universities, research laboratories and other organizations that aim to advance Lyme and tick-borne disease diagnosis and treatment, human vaccination, awareness and education, data science and management, and ecological prevention. Philanthropic funding, including a new public-private partnership around novel diagnostic technologies, is critical to address the historically small amount of federal funding for LD compared to some other infectious disease of public health concern. While the Kay Hagan Tick Act did recently boost federal support for LD research (343), more is needed.

LD is a large topic. Some important advances were excluded for space considerations, such as immune evasion by *Bb* spirochetes, common co-infections, model organisms and *in vitro* systems for the study of LD, neuronal sensitization hypothesis for the etiology of PTLD specifically and neuroborreliosis in general, among others.

In closing, in consideration of the unique global circumstances with the COVID-19 pandemic, it is important to highlight several features of the current context that may impact the LD community. For example, COVID-19 may further complicate diagnosis of LD since non-specific symptoms in these two conditions overlap and people may be spending more time outdoors. The emergence of a persistent syndrome, popularly referred to as long COVID, among a subset of patients following treatment or convalescence (344) may invigorate research and provide insights that carry-over into other infectious diseases with post-treatment sequelae, such as LD.

## Author Contributions

JRB wrote the first draft of the manuscript. BLJ, EJH, MEE, AB, and RLM wrote sections of the manuscript. All authors contributed to manuscript revision, read, and approved the submitted version.

## Conflict of Interest

AGR is a scientific advisor to NeonMind Biosciences and ETHA Natural Botanicals. In conjunction with Dr. S. Sontakke and North Carolina State University, EBB holds US Patent No. 7,115,385; Media and Methods for Cultivation of Microorganisms, which was issued on October 3rd, 2006. He is a co-founder, shareholder and Chief Scientific Officer for Galaxy Diagnostics, a company that provides advanced diagnostic testing for the detection of Bartonella spp. and Borrelia infections. RM is a co-founder, shareholder and the Chief Technical Officer for Galaxy Diagnostics. RTM is a paid consultant/speaker for Zoetis and receives license related income from Zoetis. MJS serves on the Scientific Advisory Board for the Global Lyme Alliance and has been issued a patent [US Patent No. 10 481 165] for “Elevated CCL19 after completion of therapy for acute Lyme disease identifies patients at risk for development of post-treatment Lyme disease syndrome who will benefit from further antibiotic therapy”. AO has pending patent applications on the development of Lyme disease diagnostic tests. The remaining authors declare that the research was conducted in the absence of any commercial or financial relationships that could be construed as a potential conflict of interest.

## Publisher's Note

All claims expressed in this article are solely those of the authors and do not necessarily represent those of their affiliated organizations, or those of the publisher, the editors and the reviewers. Any product that may be evaluated in this article, or claim that may be made by its manufacturer, is not guaranteed or endorsed by the publisher.

## Footnotes

^1^ https://www.columbia-lyme.org/clinical-trials-network
